# Targeting ceramide metabolism to restore hypoxia-induced apoptosis in p53-deficient colon cancer cells

**DOI:** 10.1371/journal.pone.0340295

**Published:** 2026-01-06

**Authors:** Karen Mechleb, Nancy Hourani, Maya Fakhry, Osama A. Alyamani, Rouba Hage-Sleiman, Hicham Younes, Antoine Abou Fayad, Nadia Soudani, Amer Sakr, Nadine Darwiche, Georges Nemer, Raya Saab, Marguerite Mrad, Ghassan Dbaibo

**Affiliations:** 1 Department of Biochemistry and Molecular Genetics, Faculty of Medicine, American University of Beirut, Beirut, Lebanon; 2 Department of Louvain, Institute of Biomolecular Science and Technology, Faculty of Sciences, Catholic University of Louvain, Louvain, Belgium; 3 Department of Pediatrics and Adolescent Medicine, Center for Infectious Diseases Research, Faculty of Medicine, American University of Beirut, Beirut, Lebanon; 4 Department of Toxicology, Toxicology Lab Research and Training Center, Faculty of Health Sciences, American University of Science & Technology (AUST), Beirut, Lebanon; 5 Forensic Toxicology Laboratory, Center of Forensic & Digital Sciences, Abu Dhabi Judicial Department (ADJD), United Arab Emirates; 6 Department of Biology, Faculty of Sciences, Lebanese University, Hadath, Lebanon; 7 Department of Experimental Pathology, Immunology and Microbiology, Faculty of Medicine, American University of Beirut, Beirut, Lebanon; 8 Department of Surgery, Washington University School of Medicine, St. Louis, Missouri, United States of America; 9 Division of Genomics and Translational Biomedicine, College of Health and Life Sciences, Hamad Bin Khalifa University, Doha, Qatar; 10 Department of Pediatrics, Division of Hematology/Oncology, Stanford University, Stanford, California, United States of America; 11 Department of Medicine, Division of Endocrinology, Metabolism and Lipid Research, Washington University School of Medicine, St. Louis, Missouri, United States of America; Universite Paris Diderot-Paris7 - Batiment des Grands Moulins, FRANCE

## Abstract

Hypoxic stress in solid tumors triggers growth arrest and apoptosis through p53 activation and stabilization. This environment inactivates p53 and drives the expansion of p53-mutant clones, which accentuate tumor aggressiveness. Ceramide, a signaling sphingolipid, was previously identified as a downstream collaborator with p53 in the stress-induced apoptosis and cell cycle arrest. Among sphingolipids, the balance between pro- and anti-apoptotic products, dictated by the expression and activity of appropriate enzymes, helps determine cell fate in response to hypoxia. The current study aimed to understand the role of ceramide in HCT116 human colon cancer cells response to hypoxia in the presence or absence of p53, and to determine whether the modulation of ceramide metabolism could sensitize the resistant p53-deficient cells to hypoxia-induced cell death. We observed that HCT116 p53-deficient cells were resistant to hypoxic cell death. We explored the role of ceramide in this response by screening for different sphingolipid metabolites through liquid-chromatography-mass spectrometry, and by measuring the expression of key enzymes involved in ceramide biosynthesis and breakdown. We also evaluated the changes in the cellular response to hypoxia associated with introduction of sphingolipid metabolites or with modulating the activity of related sphingolipid-metabolizing enzymes. In hypoxic p53-deficient cells, ceramide was synthesized via the *de novo* pathway through the action of ceramide synthases and dihydroceramide desaturase (DEGS1) driving the evasion of hypoxia-induced apoptosis. Among the accumulating ceramide species in p53 deficient cells, C24-ceramide was the most abundant and possibly contributing to their resistance. Tipping the sphingolipid balance in favor of pro-apoptotic sphingolipids, through the addition of C6 ceramide or sphingosine, or through the combined pharmacologic inhibition of DEGS1 and sphingosine kinase 1, helped circumvent the cellular resistance to hypoxia-induced apoptosis in cells lacking p53. Therefore, modulating sphingolipid metabolism may be a viable approach in the treatment of solid tumors with hypoxic regions.

## Introduction

Hypoxia is a complex environmental aspect of exponentially growing solid tumors that drives cancer aggressiveness, and metastasis [1,2]. Cellular stress caused by the challenging decrease in oxygen availability, due to the remoteness from supporting vasculature and the chaotic structure of tumor vascular networks, engenders DNA breaks. As a result, DNA repair systems – such as p53 – get activated, and the progression into the cell cycle is halted to allow time for repair. When the damage is unrepairable, cells undergo p53-dependent programmed cell death. Alternatively, hypoxia drives the loss of “the guardian of the genome” by inducing mutations in the *TP53* gene, and acting as an environmental positive selector for the mutant clones harboring malignant traits that allow survival in hypoxic environments [3,4].

This undesirable outcome resulting from the disabled apoptotic process prompts the development of strategies that could specifically sensitize the p53-defective clones evolving in the hypoxic regions. Some of the strategies focusing on the reconstitution of wild-type p53 include adenoviral delivery of wild-type p53, inhibition of p53 proteasomal degradation through the disruption of p53-Mdm2 interaction, and restoration of mutant p53 to wild-type p53. Despite their promise, these approaches were faced with several limitations that translated into poor clinical responses [5].

Previous studies shed light on ceramide, a signaling sphingolipid that, similarly to p53, mediates several stress-induced cellular responses such as cell cycle arrest and apoptosis [6]. However, different ceramide species, categorized by their acyl chain lengths, mediate distinct biological functions that are sometimes anti-apoptotic [7,8]. We have shown that in systems where p53 is expressed, ceramide accumulation is dependent on p53 and is generated by *de novo* synthesis [9,10]. Moreover, ceramide-induced apoptosis and cell cycle arrest can occur independently of p53 in other models [11,12]. We have also demonstrated, in a hypoxic neonatal mouse model, that dihydroceramide desaturase (DEGS1), the enzyme that catalyzes the final step of the *de novo* ceramide synthesis pathway, acts as a checkpoint in the regulation of ceramide-based responses to hypoxia [13–15]. Chronic exposure to hypoxia engendered hypertrophy of the right ventricle accompanied by a decrease in the expression of DEGS1 gene and ceramide levels, and accumulation of dihydroceramide (DhCer) [13–15]. Likewise, DEGS1 activity was inhibited, and DhCer accumulated in lung and colon cancer cells exposed to chronic hypoxia [16]. Conversely, acute hypoxia promoted ceramide production via the *de novo* pathway in SH-SY5Y neuroblastoma cells leading to ceramide-dependent apoptosis [17].

On the other hand, sphingosine-1-phosphate (S1P), a ceramide catabolite known to promote cellular proliferation and survival [6,18,19], was induced through sphingosine kinase 1 (SK1) upregulation following short periods of hypoxia, driving vasodilation of arteries [20]. Likewise, Obeid and colleagues have reported that hypoxia increased SK1 mRNA levels, protein expression, and enzyme activity, followed by intracellular S1P production and S1P release [21]. In turn, SK1 stabilized hypoxia-inducible factor (HIF-1α), a major orchestrator of the hypoxic response, through preventing its von Hippel-Lindau protein-mediated degradation by the proteasome [22].

Altogether, these findings underscore the role of DEGS1, SK1 and the DhCer/ceramide/S1P rheostat in the bioadaptive response to hypoxia. Still, a deeper understanding of the “sphingolipid scenario” in the p53-mediated and independent responses to hypoxia is needed to propose promising sphingotherapies for p53-defective tumors. In this study we investigated the role of ceramide and its metabolizing enzymes, DEGS1 and SK1, as key bioresponders to oxygen deprivation, notably in the absence of p53. We also determined whether the modulation of ceramide metabolism could restore the apoptotic response in p53-deficient cells upon incubation in oxygen-scarce environment.

## Materials and methods

### Cell culture

The human colon cancer cell line HCT116 p53^+/+^ (p53 wild-type, or p53-proficient) was purchased from the American Tissue Culture Collection, ATCC, Manassas, VA. The HCT116 p53^-/-^ cell line (p53 null, or p53-deficient) was kindly provided by Dr. Carlos Maria Galmarini, PharmaMar, Madrid, Spain. HCT116 p53^+/+^ cells express wild type p53 protein. HCT116 p53^-/-^ cells were derived from HCT116 cells by disrupting the two *p53* alleles using homologous recombination [23]. HCT116 p53^-/-^ cells were cultured in Dulbecco’s Modified Eagle’s Medium (DMEM)-high glucose, with 10% fetal bovine serum (FBS), 1% penicillin/streptomycin, 1% sodium pyruvate and 1% nonessential amino-acids. HCT116 p53^+/+^ were cultured in Roswell Park Memorial Institute (RPMI 1640), with 10% FBS, 1% penicillin/streptomycin, 1% sodium pyruvate. Cells maintained under normoxic conditions were incubated at 37°C, 5% CO_2_, and 21% O_2_ and passaged twice weekly. For the hypoxic conditions, cells were incubated for different time points in a 5% CO_2_ incubator equipped with an oxygen sensor set to 1% O_2._

### MTT assay

Cells were seeded in 96-well plates at a density of 5000 cell/well in 100 μL of FBS-supplemented medium and left to attach overnight. The following day, cells were treated with pharmacological inhibitors of sphingolipid enzymes, sphingosine, C6 DhCer or C6 ceramide before being incubated in hypoxic (1% O_2_) or normoxic (21% O_2_) conditions for periods of 24, 48 and 72 hours. The effect of hypoxia on cell viability was evaluated with the MTT (3-(4,5-dimethylthiazol-2-yl)-2,5-diphenyltetrazolium bromide) assay. 30 μL of a stock solution of MTT (5 mg/ml, Sigma) were added to 100 μL of culture medium/well, and the mixture was incubated for 4 hours at 37°C in a humidified atmosphere containing 5% CO_2_. The crystals of formazan were then dissolved with dimethyl sulfoxide (DMSO, Sigma), and reduced MTT levels were determined by measuring absorbance spectrophotometrically at 595 nm, using an ELISA microplate reader.

### Flow cytometric analysis of DNA content

Cells were seeded in 6-well plates at a density of 150,000 cells/well and left to attach overnight. In some experiments, cells were pre-treated with 2.5 μM of C6 exogenous ceramide and then incubated in hypoxic or normoxic conditions. At each time point, cells were harvested along with their supernatant. Pelleted cells were permeabilized with 70% ethanol, treated with 1% RNase, and stained with propidium iodide solution. Analysis of cells distribution in the sub G0, G0/G1, S, and G2/M phases of the cell cycle was performed on a BD FACSAria SORP flow cytometer, operated by BD FACS DIVA software. Cells less intensely stained than G1 cells (sub G0 cells) in flow cytometric histograms were considered as dead cells.

### Annexin V-propidium iodide staining

Cells were seeded in 24-well plates at a density of 30,000 cells/ well and pre-treated with 2.5 μM of C6 exogenous ceramide prior to their exposure to hypoxia for 72 hours. Cells were harvested by trypsinization and collected along with initial culture medium and wash buffer to ensure inclusion of detached cells. Cells were pelleted by centrifugation (300 g, 10 minutes, 4°C) and re-suspended in 20 µl of 1X binding buffer containing 2 µl of annexin V-FITC solution (Miltenyi Biotec) for 15 minutes in the dark at room temperature. Subsequently, cells were washed then resuspended in 100 μL of 1X binding buffer containing 1µl of propidium iodide solution. Cells were acquired on Guava® EasyCyte 8, Millipore flow cytometer, and results were analyzed by GuavaSoft™ 2.7 Software. Alive cells are negatively stained for both Annexin V and PI. Early apoptotic cells are Annexin V positive and PI negative. Late apoptotic cells are both Annexin V and PI positive. Necrotic cells are Annexin V negative and PI positive.

### Liquid chromatography mass spectrometry (LC/MS)

Cells were seeded in T25 flasks at a density of 500,000 cells/ flask, and subsequently grown in hypoxia or normoxia. At indicated time points, cells were scraped, harvested along with their supernatant, and washed with PBS. Extracellular medium was collected when needed. Lipids were extracted following the method described by Bielawski *et al.* [24] and Bligh *et al.* [25]. Sphingolipid levels were quantified by LC/MS using the API 4000 LC-MS/MS triple-stage quadrupole mass spectrometer operating in a multiple reaction monitoring positive ionization mode (Fig 2A and Fig 4A) and with LC-ESI-MS/MS based on an AB Sciex X500R QTOF ESI mass spectrometer (SCIEX, Framingham, MA, USA) (Fig 4C and Fig 5A). The ceramide/sphingolipid internal standard (IS) mixture I (LM6002, Avanti polar lipids) was used for internal calibration. Levels of ceramide and sphingosine were normalized to protein cellular content and expressed in picomoles per nanogram of protein. Levels of total ceramide and S1P were normalized to inorganic phosphate content and expressed in picomolar (pM) per picomolar (pM) of inorganic phosphate.

**Fig 1:**
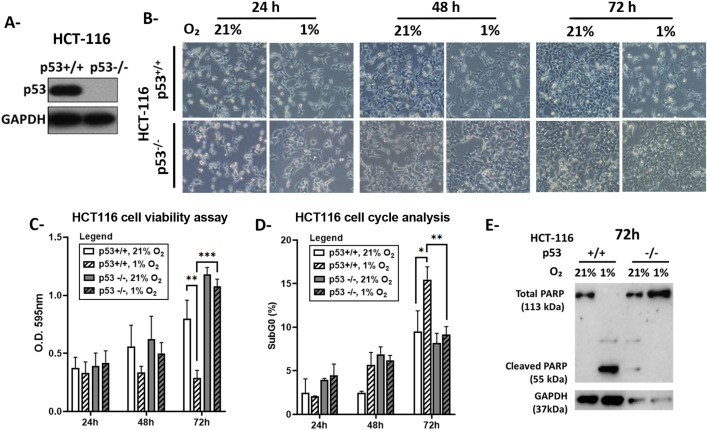
Response of HCT116 p53^+/+^ and p53^-/-^ cells to hypoxia. A- Protein levels of p53 were determined by western blot. GAPDH was used as a loading control. B- Cell morphology was observed in normoxia (21% O_2_) or hypoxia (1% O_2_) using phase-contrast microscopy. Images were captured at 100 × magnification and are representative of three independent experiments. C- Cell viability was measured by MTT assay on HCT116 p53^+/+^ (white) and p53^-/-^ (grey) cells after 24, 48 and 72 hours of incubation in normoxia (plain) or hypoxia (patterned). The optical density (O.D.) was measured at 595 nm. Values represent the average of three independent experiments ± **S.**D. D- Percentage of cells in sub-G0 phase of the cell cycle was measured by flow cytometry. Each column represents the mean ± **S.**D. of two independent experiments. * p < 0.05, **p < 0.01, *** p < 0.001. E- Analysis of PARP cleavage by western blot. GAPDH was used as a loading control. Blots are representative of two independent experiments.

**Fig 2:**
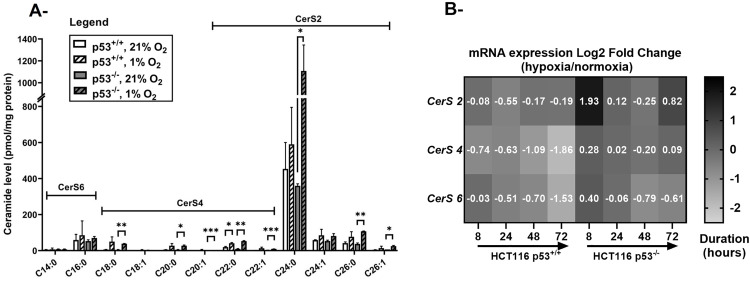
Ceramide level and ceramide synthase expression in HCT116 p53^+/+^ and p53^-/-^ cells exposed to hypoxia. A- Levels of ceramide species at 72 hours post-hypoxia. HCT116 p53^+/+^ and p53^-/-^ cells were incubated in 21% and 1% O_2_ and lipids were extracted. Ceramide species were quantified by LC/MS and normalized to protein content (pmol ceramide/mg protein). Values represent the average of two independent experiments ± **S.**D. *p < 0.05, **p < 0.01. B- mRNA expression levels of ceramide synthases (*CerS2*, *CerS4*, and *CerS6*) were assessed by qRTPCR, normalized to *β-actin* expression and represented as log_2_ fold change (hypoxia/normoxia). Values represent the average of two independent experiments.

**Fig 3:**
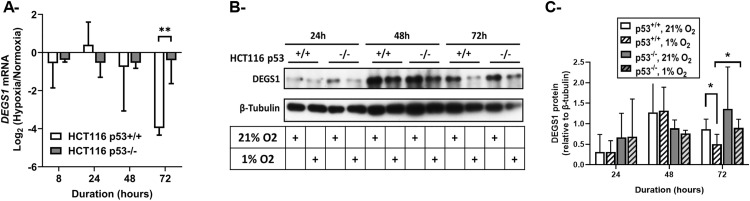
DEGS1 expression in HCT116 p53^+/+^ and p53^-/-^ cells exposed to hypoxia. A- mRNA expression of DEGS1 was assessed by qRT-PCR after 24, 48 and 72 hours of hypoxia, normalized to β-actin expression and represented as log_2_ fold change (hypoxia/normoxia). Values represent the average of three independent experiments ± **S.**D. B- DEGS1 protein levels were assessed by western blot after 24, 48 and 72 hours of hypoxia. β-tubulin was used as a loading control. C- Quantification of DEGS1 band intensity was performed by ImageJ and normalized to that of β-tubulin. Values represent the average of three independent experiments ± **S.**D. on 24 and 48 hours and of five independent experiments ± **S.**D. on 72 hours. *p < 0.05; **p < 0.01.

### Quantitative real-time PCR (qRT-PCR)

Cells were subjected to either hypoxia or normoxia for 8, 24, 48 and 72 hours. Cells were collected at the indicated time points, and total RNA was extracted using the RNA isolation kit “RNeasy Mini Kit” (Qiagen) as recommended by the manufacturer. The cDNA was synthetized by reverse transcription using QuantiTech Reverse Transcription Kit (Qiagen) as per the manufacturer’s instructions. Quantitative real-time PCR (qRT-PCR) was performed in duplicates in 96-well plates using Sybr Green (SYBR Green JumpStart Taq ReadyMix, Sigma) and CFX96 Real-Time PCR detection system (Bio-Rad). Ct values were then computed and normalized to β-actin values to determine the relative changes in mRNA expression of *CerS2*, *CerS4*, *CerS6*, *DEGS1* and *SK1* genes. The primers used are listed in Table 1.

**Table 1:** Sequences of forward and reverse primers.

Gene	Forward primer (5’-3’)	Reverse primer (5’-3’)
*β-actin*	CTG GCA CCA CAC CTT CTA CA	AGC ACA GCC TGG ATA GCA AC
*CerS2*	CCG ATT ACC TGC TGG AGT CAG	GGC GAA GAC GAT GAA GAT GTT G
*CerS4*	CTT CGT GGC GGT CAT CCT G	TGT AAC AGC AGC ACC AGA GAG
*CerS6*	ACA TTC CTT CAG CCT CCT GGA GTT	GCT CCC TGG TTT CCA GGC CAC
*DEGS1*	CCA ACA TTC CTG GAA AAA GTC TTC	GCC TCT TCA TTC TTG AGT AGG GA
*SK1*	CGC CGC AGG GAA TGA CAC C	GCC TGT CCC CCC AAA GCA TAA C

### Western blot analysis

Cells were seeded in T25 flasks at a density of 500,000 cells/flask and incubated in hypoxic conditions for 24, 48 and 72 hours. At each time point, cells were scraped and pelleted along with their supernatant. Proteins were extracted using Laemmli buffer and quantified using the Bio-Rad dye-binding assay as described by the manufacturer. The concentrations of proteins in the samples were calculated using different concentrations of BSA as standards. 30 µg of proteins were resolved on polyacrylamide gel and electro-transferred onto polyvinylene difluoride (PVDF) membranes (Bio-Rad). Immunoblotting was performed at 4°C overnight using primary anti-p53 (sc-126, Santa Cruz Biotechnology, dilution: 1:200), anti-PARP (ab191217, Abcam, dilution: 1:100) and anti-DEGS1 (SAB2100559, Sigma-Aldrich, dilution: 1:200) antibodies. Appropriate horseradish peroxidase-conjugated secondary antibodies (dilution: 1:5000) were subsequently added, and protein bands were visualized through the ChemiDoc MP imaging System (Bio-Rad) using Clarity™ ECL Western Blotting Substrate (Bio-Rad). Bands were quantified using ImageJ software (NIH, USA), and protein expression was normalized to that of β-tubulin (SAB4200715, dilution: 1:500) or GAPDH (ab8245, Abcam, dilution: 1:1000).

### Statistical analysis

Statistical analyses were performed using GraphPad Prism 8.0.1 software (GraphPad Software, San Diego, CA). *P* values were calculated using the unpaired Student’s t-test and differences were considered statistically significant at *P* < 0.05 (* *P* < 0.05; ** *P* < 0.01; *** *P* < 0.001).

## Results

### HCT116 cells resist hypoxia-induced apoptosis in the absence of p53

The differential expression of p53 in HCT116 cells was validated by western blot. HCT116 p53^+/+^ cells express p53, unlike HCT116 p53^-/-^ cells that showed no p53 protein expression (Fig 1A).

To determine the role of p53 in the cellular response to hypoxia, cells were incubated in 1% O_2_ for 24, 48 and 72 hours. Phase contrast microscopy examination showed that the cellular density decreased in hypoxia in HCT116 p53^+/+^ cells with no significant change in morphology (Fig 1B). MTT results showed that, by 48 hours, the viability of HCT116 p53^+/+^ cells dropped with the decrease becoming significant at 72 hours of exposure to hypoxia (Fig 1C), concomitant with the observed reduction in cell density (Fig 1B). In contrast, upon incubation in 1% O_2_, p53-deficient cells preserved their viability, and reached a density similar to control cells grown at 21% O_2_ (Figs 1B and 1C). To assess whether the decrease in viability is driven via the induction of cell cycle arrest or cell death rather than just a decrease in the metabolic activity of the cell, we examined the cell cycle distribution in HCT116 cells exposed to hypoxia. Unlike HCT116 p53^-/-^ cells, p53^+/+^ cells showed a significant increase in the percentage of sub-G0 cells (Fig 1D) indicating DNA fragmentation, accompanied by PARP cleavage (Fig 1E) after 72 hours of exposure to hypoxia. Accordingly, these results support an apoptotic response to hypoxia in HCT116 p53^+/+^ cells and indicate that p53 is required for the apoptotic response to hypoxic stress whereas defective p53 expression conveys resistance to hypoxia-induced apoptosis.

### Hypoxia modulates ceramide metabolism in HCT116 cells

To define the role of ceramide in the response of HCT116 cells to hypoxia, we carried out a quantitative analysis of sphingolipids in cellular lipid extracts by liquid chromatography mass spectrometry (LC/MS). Significant changes in ceramide levels were only reported at 72 hours post-hypoxia (Fig 2A) (data for 24 and 48 hours are shown in supplementary figure S1 Fig). Long (C18:0), very long (C20:0, C20:1, C22:0, C22:1 and C24:0), and ultra-long (C26:0 and C26:1) chain ceramides were significantly accumulated in p53^-/-^, with C24:0 ceramide being the most abundant (Fig 2A). Only the level of C22:0 ceramide was increased significantly in HCT116 p53^+/+^ cells 72 hours post-hypoxia.

We validated the sphingolipidomic profile by assessing the gene expression of ceramide synthases (CerS) 2, 4 and 6. CerS4 and CerS2 are known to produce C18-C22 and C22-C26 ceramides, respectively [26,27]. *CerS2* and *CerS4* expression levels were found to decrease in p53^+/+^ cells, notably by 72 hours of exposure to hypoxia but were either sustained or slightly increased in p53-deficient cells when compared to levels in normoxic cells (Fig 2B). CerS6, responsible for C14 and C16 ceramide species generation, was downregulated in hypoxic HCT116 cells regardless of p53 status (Fig 2B). Notably, the levels of its corresponding ceramides (C14:0 and C16:0) were not significantly altered following the drop in oxygen tension (Fig 2A).

The above data suggested a potential role for CerS 2 and 4 in the ceramide accumulation observed in HCT116 p53^-/-^ cells upon exposure to hypoxia. Ceramide synthases carry out the N-acylation of sphingoid bases either generated from serine and palmitoyl CoA condensation via the *de novo* biosynthetic pathway or recycled from complex sphingolipids through the salvage pathway. DEGS1 enzyme catalyzes the final step of *de novo* ceramide synthesis by converting DhCer to ceramide. Due to its electron transfer activity, DEGS1 has long been studied as a major orchestrator of sphingolipid-based responses to reductive environments such as hypoxia. We have previously determined, in a p53-expressing system, that chronic hypoxia promotes DhCer accumulation and halts ceramide responses through DEGS1 downregulation [13]. Therefore, we exposed p53-deficient and p53-proficient HCT116 cells to hypoxic conditions and measured DEGS1 expression. We found that, in the presence of p53, chronic hypoxia prominently decreased DEGS1 expression at the levels of mRNA (Fig 3A) and protein (Fig 3B and 3C). However, *DEGS1* expression was sustained in p53^-/-^ cells. Hence, p53 potentially inhibits ceramide accumulation in chronic low-oxygen stress by disrupting the *de novo* cascade of ceramide synthesis.

**Fig 4:**
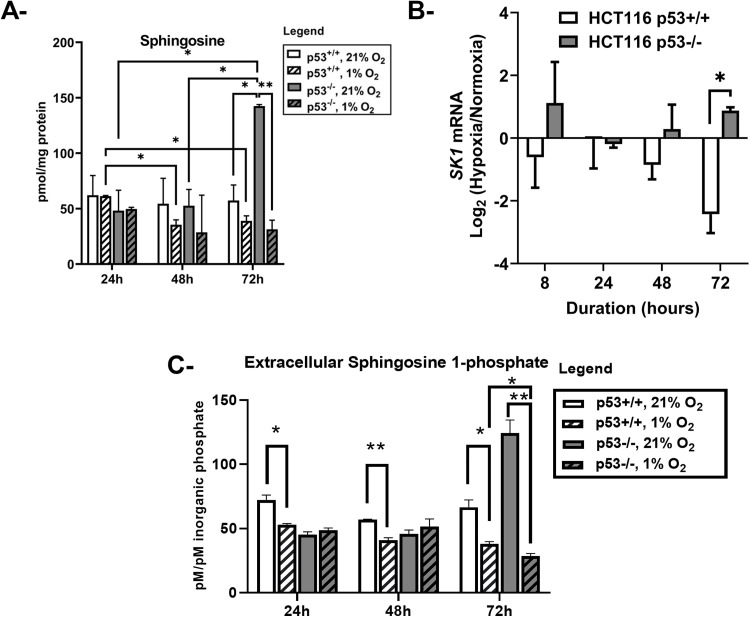
Sphingosine level and SK1 mRNA expression in HCT116 p53^+/+^ and p53^-/-^ cells exposed to hypoxia. A- Sphingosine was quantified by liquid chromatography-mass spectrometry and normalized to protein content (pmol sphingosine/mg protein). Values represent the average of two independent experiments ± **S.**D. B- mRNA expression of SK1 was assessed by qRT-PCR after 24, 48 and 72 hours of hypoxia, normalized to β-actin expression and represented as log_2_ fold change (hypoxia/normoxia). C- Extracellular medium was collected and the S1P content was quantified by LC/MS and normalized to the inorganic phosphate levels (pM S1P/ pM inorganic phosphate). Values represent the average of two independent experiments ± **S.**D. *p < 0.05, **p < 0.01.

We then investigated whether the ceramide generated through chronic exposure to hypoxia in p53^-/-^ cells was being further catabolized into the pro-survival lipid sphingosine 1-phosphate (S1P), possibly allowing the cell to resist apoptosis [21]. We measured the levels of sphingosine, the precursor of S1P, and found that in normoxia, p53^+/+^ cells maintained a steady level of sphingosine when cultured for 72 hours whereas p53^-/-^ cells showed a more than two-fold increase in sphingosine levels at 72 hours compared to baseline at 24 hours and to p53^+/+^ cells at 72 hours (Fig 4A). In hypoxia, we observed a significant decrease in the sphingosine levels in p53^+/+^ cells at the two time points 48 and 72 hours compared to their levels at 24 hours. In addition, these levels dropped significantly in p53^-/-^ cells at 72 hours (Fig 4A) accompanied by *SK1* mRNA upregulation (Fig 4B) indicating that sphingosine is possibly being converted to S1P, which may be contributing to the survival of hypoxic p53^-/-^ cells. In contrast, *SK1* mRNA levels were decreased in hypoxic p53^+/+^ cells, thus keeping sphingosine levels high and S1P levels low, indicating a possible contribution to cell death under these conditions. While we were not able to assess S1P cellular levels through LC/MS possibly because of S1P secretion outside the cell, we found that S1P extracellular levels remain steady at 24 and 48 hours, compared to the drop seen in p53 + / + cells, but are then downregulated in p53-/- cells after 72 hours of hypoxia compared to normoxia (Fig 4C).

To further elucidate the role of the different pathways of ceramide biosynthesis and biodegradation in the p53-mediated and p53-independent responses to chronic hypoxia, we treated HCT116 cells with inhibitors of ceramide-metabolizing enzymes and evaluated hypoxia-induced ceramide accumulation by LC/MS, and changes in cell viability by MTT.

**Fig 5:**
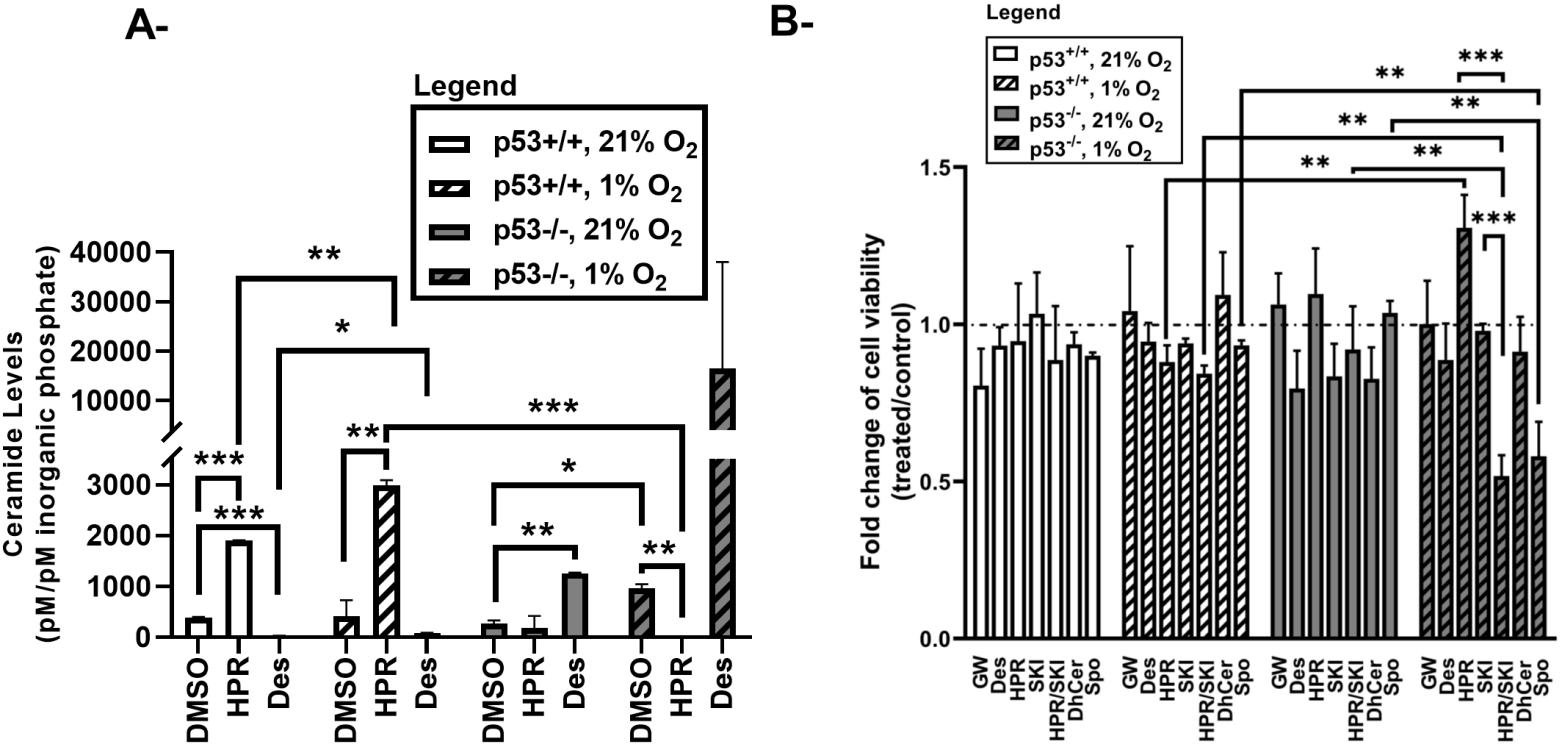
Effect of inhibitors on ceramide levels and cell viability of HCT116 p53^+/+^ and p53^-/-^ cells exposed to chronic hypoxia. A- Total ceramide levels at 72 hours post-hypoxia was measured by LC/MS and normalized to inorganic phosphate levels (pM of ceramide/ pM of inorganic phosphate). Values represent the average of two independent experiments ± S.D. *p < 0.05; **p < 0.01; *** p < 0.001. B- Cell viability was measured by MTT assay. Data are represented as fold change (treatment/DMSO control). HCT116 cells were treated with 1.5 mM of GW4869 (GW), 10 μM desipramine (Des), 10 μM 4-HPR (HPR), 10 μM SK1-I (SKI), 20 μM C6 DhCer or 20 μM sphingosine prior to their incubation in a hypoxic chamber for 72 hours. Values represent the average of three independent experiments ± S.D. **p < 0.01.

In normoxic conditions, inhibition of acid sphingomyelinase (ASMase)-induced ceramide generation using desipramine decreased ceramide levels in p53^+/+^ but did not alter their viability. However, in p53^-/-^ cells desipramine treatment increased ceramide accumulation through a pathway independent of acid sphingomyelinase without altering their cell viability. After 72 hours of exposure to hypoxia, ceramide was found to accumulate in both HCT116 models, but a more prominent increase was observed in p53^-/-^ cells as compared to p53^+/+^ cells (Fig 5A). DEGS1 and ASMase are central enzymes in the *de novo* and salvage pathways of ceramide synthesis that can be inhibited using fenretinide (4-HPR) and desipramine, respectively [28–30]. Treatment of HCT116 cells with 4-HPR decreased the ceramide levels in HCT116 p53^-/-^ cells, but not p53^+/+^ cells, in hypoxia (Fig 5A). While, desipramine treatment did not significantly alter the ceramide levels in response to hypoxia in both HCT116 models. Thus, we can infer that in the absence of p53, ceramide accumulation in response to hypoxia is DEGS1-dependent and ASMase-independent, indicating a role for *de novo* pathway of ceramide synthesis in this setting.

To determine whether ceramide accumulation impacted cell fate, we evaluated the effects of several inhibitors of sphingolipid pathway enzymes on cell viability. Treatment of HCT116 cells grown in hypoxic conditions with GW4869 or desipramine, respective inhibitors of neutral sphingomyelinase and ASmase, did not affect the cell viability (Fig 5B) and apoptotic response (data shown in S2 Fig) in both p53^+/+^ and p53^-/-^ models. To study whether DhCer accumulation affects cell viability, we treated cells with exogenous C6 DhCer but this did not influence their viability (Fig 5B). We also examined whether S1P, generated by the activity of SK1 on sphingosine, played a role in the enhanced survival of HCT116 cells. SK1 inhibition alone using SK1-I did not alter the viability in either of HCT116 cellular models (Fig 5B). Interestingly, combined inhibition of DEGS1 and SK1, markedly reduced cellular viability of p53^-/-^ cells compared to normoxic p53^-/-^ cells or to hypoxic p53^+/+^ cells. This was not observed when either enzyme was inhibited alone. This indicated that the ceramide species accumulated in p53^-/-^ through the *de novo* pathway in response to hypoxia are contributing beside the low sphingosine levels to the resistance of these cells. Therefore, to sensitize these cells we need to inhibit the *de novo* ceramide synthesis and to block SK1 from converting the existing pool of sphingosine into S1P.

Treating HCT116 cells with the ceramide catabolite, sphingosine, sensitized p53^-/-^ but not p53^+/+^ cells to hypoxia causing a decrease in cell viability. Unlike S1P, sphingosine is known to mediate antiproliferative and proapoptotic activities [31]. Altogether these data indicate that in the absence of p53, ceramide metabolic pathways control cellular responses to hypoxia by driving ceramide synthesis through the *de novo* pathway and by maintaining a low level of sphingosine. The resulting metabolic alterations in sphingolipids shape the cellular fate in response to hypoxia depending on p53 expression.

**Fig 6:**
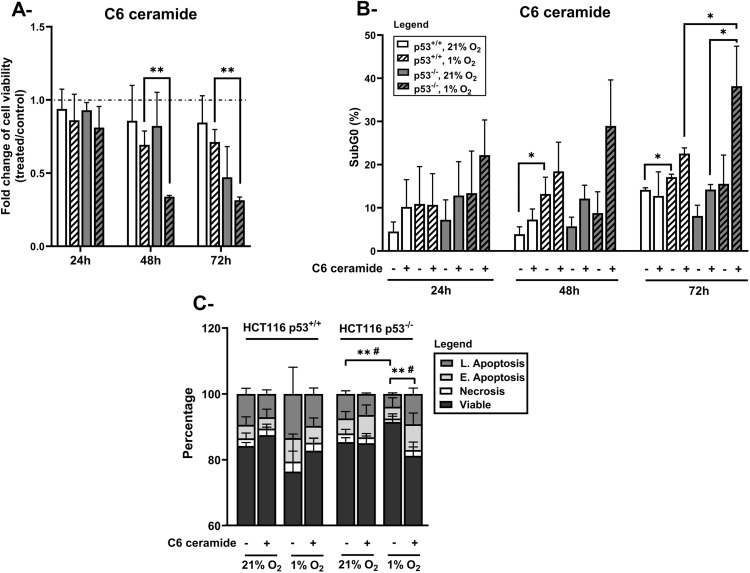
Effect of C6 ceramide on the response of HCT116 p53^+/+^ and p53^-/-^ cells to hypoxia. Cells were pre-treated with 2.5 µM exogenous C6 ceramide and exposed either to normoxia (21% O_2_) or hypoxia (1% O_2_) for 24, 48 and 72 hours. A- Cell viability was measured by MTT assay. Data are calculated as fold change (treatment/control) and represent the mean ± S.D of three independent experiments. B- Percentage of SubG0 cells was determined by flow cytometry following propidium iodide staining. Analysis was performed using BD FACSDiva™ software. Each column represents the mean ± S.D. of three independent experiments. Values represent the average of three independent experiments. *p < 0.05; **p < 0.01. C- Apoptosis was evaluated by Annexin-V-FITC/ PI flow cytometry analysis after 72 hours of incubation in either normoxia or hypoxia. Analysis was performed using Guava EasyCyte software. Values represent the average of three independent experiments. **p < 0.01 (significance between L. Apoptosis populations); #p < 0.05 (significance between viable populations). E. apoptosis: early apoptosis, L. apoptosis: late apoptosis.

Finally, we tried to sensitize HCT116 p53^-/-^ cells to hypoxia-induced apoptosis by introducing exogenous C6 ceramide. Synthetic cell permeable short chain ceramides, notably C6 ceramide, have been extensively utilized for their putative anti-cancer properties, alone or in combination with conventional chemotherapies [32–38]. When administered to the cell, short chain ceramides get deacylated and longer fatty acid chains are subsequently added to the sphingosine backbone, in order to generate endogenous longer chain ceramides [39]. We examined whether adding C6 ceramide would help overcome the resistance of HCT116 p53^-/-^ cells to hypoxia-induced apoptosis. We observed a decrease in the cellular viability in both p53^+/+^ and p53^-/-^ cells when C6 ceramide was administered before incubation in hypoxic conditions for 48 and 72 hours (Fig 6A). Interestingly, this decrease was significantly more prominent in p53^-/-^ cells as compared to p53^+/+^ cells and accompanied by an increase in apoptosis, as evidenced by cell cycle distribution (Fig 6B) and annexin V/PI staining (Fig 6C). Thus, similarly to sphingosine, C6 ceramide sensitized p53-deficient cells to hypoxia-induced apoptosis.

## Discussion

A detailed understanding of the molecular mechanisms and the metabolic pathways engaged in the p53-dependent and -independent responses to cellular stress provides further insights on how to potentially target p53 clonal mutants that are known to contribute to the resistance of many cancers to conventional therapies. Ceramide is a putative “signaling lipid” that, similarly to p53, induces apoptosis and cell cycle arrest in response to cellular stress. We have previously shown in Molt-4 leukemia cells that ceramide acts downstream of p53 and mediates the apoptotic response to γ-irradiation or treatment with actinomycin D [9,12]. Other reports described p53-independent ceramide generation under stressful stimuli such as genotoxins and ionizing radiation and linked the produced ceramide with cell death [40–42]. Here we demonstrate that in chronic hypoxia, several ceramide species accumulate in the absence of p53 (Fig 2) associated with an enhanced cellular survival (Fig 1), with the most abundant ceramide being C24:0. Knowing that ceramides harbor fatty acids with different chain lengths, several studies have indicated that some biological functions of ceramides are chain-length dependent [8,43]. While C16 ceramide accumulation promoted apoptosis in HeLa cells exposed to irradiation, the overexpression of C24:0 and C24:1 ceramides resulted in anti-apoptotic effects [43]. Similarly, C24 ceramide promoted gallbladder cancer cell proliferation and was associated with metastasis and poor prognosis [44]. In a study performed on MCF-7 and HCT116 cells, overexpression of CerS4 and 6, responsible for long chain ceramides synthesis (C16:0, C18:0 and C20:0), inhibited cell proliferation and promoted apoptosis. In contrast, overexpression of CerS2 and the resulting increased synthesis of very long chain ceramides including C24 ceramide, supported cell proliferation [8]. Hence, the hypoxia-induced stress in cells lacking p53 may promote the accumulation of very long chain ceramides synthesized by CerS2, notably C24:0.

Previous studies examining the roles of sphingolipids in the hypoxic response focused on the desaturation of the ceramide precursor, DhCer. In chronic hypoxia, Devlin *et al.* reported a shift from ceramide to DhCer in mammalian cells and in the lungs of hypoxic rats [16]. These findings were concordant with our earlier observations showing similar alterations in the Cer/DhCer balance in the heart of mice or rats exposed to chronic hypoxia [13–15]. Compared to mice raised in atmospheric oxygen concentration, hypoxic animals demonstrated a decrease in C16 ceramide levels and an increase in its precursor C16 DhCer in the right ventricle [15]. This was accompanied by the downregulation of DEGS1 in the right ventricle between weeks 4 and 8 of exposure to hypoxia as compared to normoxic mice [13]. We proposed an identical adaptation mechanism to low oxygen in rats, where the loss of ceramide was associated with cardiomyocyte proliferation and subsequent hypertrophy of the right ventricle [14]. Tipping the Cer/DhCer balance in favor of DhCer through DEGS1 downregulation was observed in reductive environments such as hypoxia. Alsanafi *et al*. observed that DEGS1 polyubiquitination and subsequent proteasomal degradation following treatment with antioxidants and phenolic compounds enhanced the DhCer response at the expense of ceramide and promoted cell survival [45]. As for the role of p53 in this setting, several studies have evaluated the p53-DEGS1 interplay under stress conditions, but their crosstalk in oxygen-defective atmosphere remains to be clarified. In our model, when p53 was expressed, hypoxia suppressed enzymes critical for *de novo* synthesis of ceramide such as DEGS1 (Fig 3) and selected ceramide synthases (Fig 2B), but ceramide levels were stable (Fig 2A). In the absence of p53, sustained expression of DEGS1 and ceramide synthases was accompanied by ceramide accumulation and correlated with cellular resistance to apoptosis (Figs 1–3). One hypothesis that could explain these observations is that DEGS1 suppression in hypoxic conditions is mediated by p53. Since p53 is a transcription factor, one could postulate the transcriptional repression of *DEGS1* gene by p53 as a potential mechanism. Solid evidence for the physical interaction between p53 and *DEGS1* promoter can be investigated through electrophoretic mobility shift assay, or chromatin immunoprecipitation studies. An indirect inverse correlation between p53 and DEGS1 was described by the group of Pyne [46]. They observed that, whereas DEGS1 expression and enzymatic activity were suppressed following SK1/2 inhibition using SKi or ABC294640, p53 expression was induced. Also, treatment with DEGS1 inhibitor, 4-HPR upregulated p53 in LNCaP-AI prostate cancer cells [46]. This duality suggests that p53 and DEGS1 may reciprocally influence each other. Further insights into the molecular mechanisms underlying the crosstalk between p53 and DEGS1 in cellular stress should be pursued to explore the possibility of the development of effective therapies targeting this axis.

While DEGS1 acts as a terminal enzyme in the *de novo* cascade of ceramide production, ceramide synthases partake in both the *de novo* synthesis of ceramide, and the re-acylation of the free sphingosine recycled through the action of acid sphingomyelinase in lysosomal compartments, namely the salvage pathway [47–49]. The decrease in hypoxia-induced ceramide accumulation in p53^-/-^ but not p53^+/+^ cells following DEGS1 inhibition (Fig 5A) provides further evidence for the role of the *de novo* pathway in p53-independent ceramide generation in oxygen deprivation. Kang *et al.* have previously shown that ceramide synthesized through the *de novo* pathway upon hypoxia in SH-SY5Y neuroblastoma cells, and that ceramide synthase inhibition using fumonisin B1 reduced ceramide levels in hypoxic environments [17]. In addition, in NT-2 neuronal cells subjected to hypoxia/reoxygenation, ceramide accumulated *via* the salvage pathway, primarily through the action of CerS5 (LASS5) and ASMase [50]. In these studies, ceramide production in oxygen-deprived environment was linked to apoptosis. In HCT116 cells, hindering ceramide synthesis through ASMase inhibition using desipramine did not alter cell fate in hypoxia irrespective of p53 status (Fig 5B). Enzymatic inhibition of DEGS1 yields two metabolic consequences, the drop in ceramide levels and the accumulation of DhCer. Although formerly deemed inert, various studies underscored the biological activities of DhCer ranging from the induction of autophagy, apoptosis, or cell cycle arrest to promoting cell proliferation [14,51]. When administered to the cell, exogenous DhCer gets deacylated and long chain fatty acids are added to the sphinganine backbone yielding long chain DhCers. Treatment with DhCer did not alter the cellular viability in hypoxia (Fig 5B) indicating that ceramide by itself, and not DhCer, controls cell fate in p53-defective model challenged with low oxygen. This finding contrasts with the role that we previously attributed to DhCer as a driver of cell proliferation and hypertrophy [14,17,50].

In hypoxia, SK1 was overexpressed in p53^-/-^ cells compared to p53^+/+^ cells, accompanied by a significant drop in sphingosine levels, the precursor of S1P. It is noteworthy to mention that the basal level of sphingosine, assessed in normoxic conditions, was significantly higher in p53^-/-^ cells when compared to their p53^+/+^ counterparts. This can be explained by the finding that, in the absence of p53, HCT116 cells demonstrated an increased metabolic activity. Thus, as the cells approach confluency, they tend to recycle sphingolipids, possibly resulting in increased sphingosine production. Our findings are in line with previous evidence that tightly linked SK1 to an improved physiological adaptation to hypoxia in different cellular and animal models [52–54]. Ader *et al.* demonstrated in a series of cell lines that SK1 expression is triggered by hypoxia; in its turn, SK1 promotes HIF-1α and HIF-2α stabilization by preventing their von Hippel-Lindau protein–mediated proteasomal degradation [22,55]. It was previously demonstrated that hypoxia not only activates SK1 and stimulates S1P production from sphingosine, but also triggers S1P release [21]. It is also worthy to mention that Ceramidases are involved in controlling the balance between S1P and ceramides/sphingosine levels. This group of enzymes can hydrolyze ceramide into sphingosine and act in parallel with, or as a compensatory mechanism to, ceramide synthase activity [56,57]. However, sphingosine and S1P extracellular levels were downregulated in p53^-/-^ cells in response to hypoxia. This finding might suggest S1P is unlikely to be contributing to the sustained resistance (72 hours) of p53-deficient cells to hypoxia-induced apoptosis although it may do so in the short term, the first 48 hours, where the balance with pro-apoptotic sphingolipids may favor survival. The pattern (observed at 72 hours) suggests that the conversion of ceramide to sphingosine is impaired indicating that ceramidase activity may be reduced or inhibited. We postulate that the sustained resistance of these cells to hypoxia-induced apoptosis could be driven by anti-apoptotic ceramide species and low sphingosine levels. In addition, we speculate that SK1 upregulation in p53^-/-^ cells may be occurring as a feedback mechanism to restore S1P levels. Exogenous S1P acts as ligand for five G protein coupled receptors S1PR1-5. S1PR1 was shown to be upregulated after exposure to hypoxia, and its knockdown and functional antagonism markedly reduced HIF2-α expression implying a tight link between hypoxia and the exogenous S1PR-mediated signaling [55]. This pathway should be evaluated in future experiments.

Finally, we demonstrated the potential of exogenous C6 ceramide administration to selectively suppress the growth of p53-deficient cells and sensitize them to hypoxia-induced apoptosis. As it reaches the inner cellular compartments, de-acylation and re-acylation reactions of exogenous ceramide replace the short 6C fatty acid with a longer chain [39,58]. C6 ceramide promise in cancer therapy has been extensively described in preclinical models alone or in combination with various chemotherapeutic drugs including doxorubicin, paclitaxel (Taxol), histone deacetylase inhibitor, vincristine, pemetrexed, and others [34–38,59]. Moreover, C6 ceramide significantly suppressed the proliferative and metastatic capacity of gallbladder cancer cells by competing with C24- ceramide and abrogating its oncogenic activity [44].

## Conclusion

This study demonstrates that ceramide *de novo* synthesis is activated under hypoxic conditions in p53-deficient cells, primarily through the coordinated action of CerS2, CerS4, and DEGS1. The resulting accumulation of C24 ceramide and reduction in sphingosine levels establish an anti-apoptotic signature, highlighting the role of sphingolipid rheostats including the Ceramide: S1P, DhCer:Ceramide, and Sphingosine:S1P ratios in regulating cell fate under stress. Importantly, we show that exogenous C6 ceramide selectively suppresses the growth of p53-deficient cells and sensitizes them to hypoxia-induced apoptosis.

While these results provide a strong mechanistic and translational foundation, *in vivo* validation and assessment of potential toxicity are warranted. Future studies should explore combination therapies, delivery strategies, and the broader applicability of ceramide modulation across diverse tumor types.

**Fig 7:**
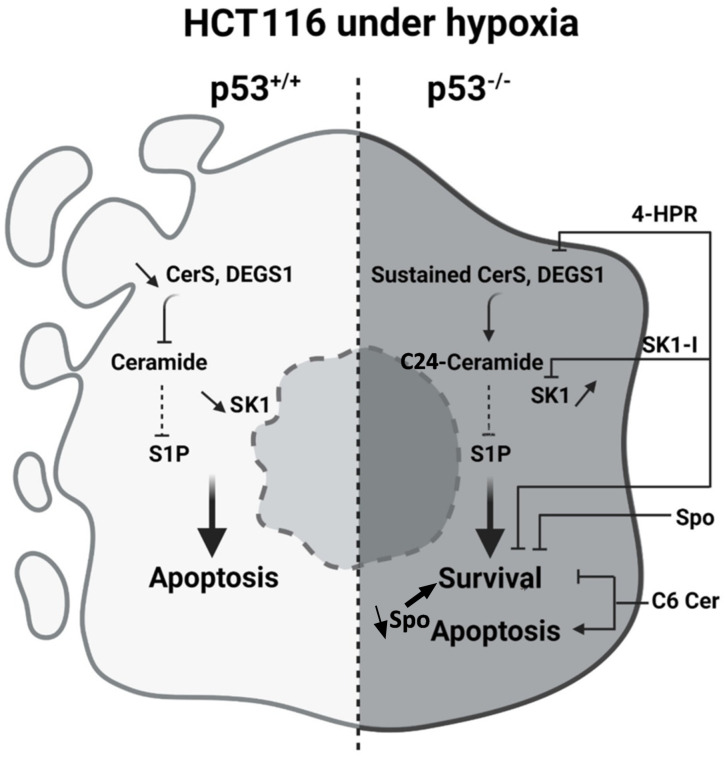
Modulation of ceramide metabolism in HCT116 cells in response to hypoxia. In p53^+/+^ cells, hypoxia downregulates CerS2, 4, 6 and DEGS1. It also inhibits SK1 leading to apoptosis. However, in the absence of p53, CerS2 and 4 expression is sustained along with that of DEGS1. As a result, different ceramide species accumulate, particularly long and very long chain ceramides, of which C24:0 ceramide is the most abundant and promoting beside the low sphingosine levels to cell survival in response to oxygen deficit. Combined inhibition of DEGS1 and SK1 or treatment of HCT116 cells with sphingosine or C6 ceramide typically restored p53-like functions in p53-deficient cells exposed to hypoxia and sensitized these cells to hypoxia-induced apoptosis.

In summary, modulating ceramide metabolism represents a promising avenue to selectively target hypoxic, p53-deficient tumors and overcome chemoresistance, with clear implications for the development of novel therapeutic strategies. Findings from the present study are summarized in Fig 7.

## Supporting information

S1 Fig Changes in the levels of medium, long, very long and ultralong chain ceramide species in HCT-116 cells subjected to hypoxia. Different molecular species of ceramide were quantified by liquid chromatography-mass spectrometry and normalized to protein content (pmol ceramide/mg protein). Data is the average of two independent experiments ± S.D. *p < 0.05; **p < 0.01; ***p < 0.001.
(TIF)

S2 Fig Evaluation of the effect of GW4869 and Desipramine on the response of HCT-116 cells to hypoxia. Fold change of the % of apoptotic cells in the GW4869-treated cells/control or in the Desipramine-treated cells/control. The percentage of cells in the subG0 phase of the cell cycle was quantified by flow cytometry. Values represent the ratios of the percentages obtained from GW4869-treated cells or Desipramine-treated cells over their vehicle-treated control. Each column represents the mean ± S.D. of the ratios calculated from three independent experiments.
(TIF)

S3 Fig DEGS1 expression in HCT116 p53^/+^ and p53^-/-^ cells exposed to hypoxia. DEGS1 protein levels were assessed by western blot after 24, 48 and 72 hours of hypoxia. β-tubulin was used as a loading control (additional blot).
(TIF)

S1 File Raw dataset of all graphs. All relevant data in this study have been deposited in this file.
(XLSX)
