# Supplementary figures and images for: Targeting ceramide metabolism to restore hypoxia-induced apoptosis in p53-deficient colon cancer cells
Source: PLoS One. 2026 Jan 6;21(1):e0340295. doi: 10.1371/journal.pone.0340295 (PMC12773810; doi:10.1371/journal.pone.0340295)

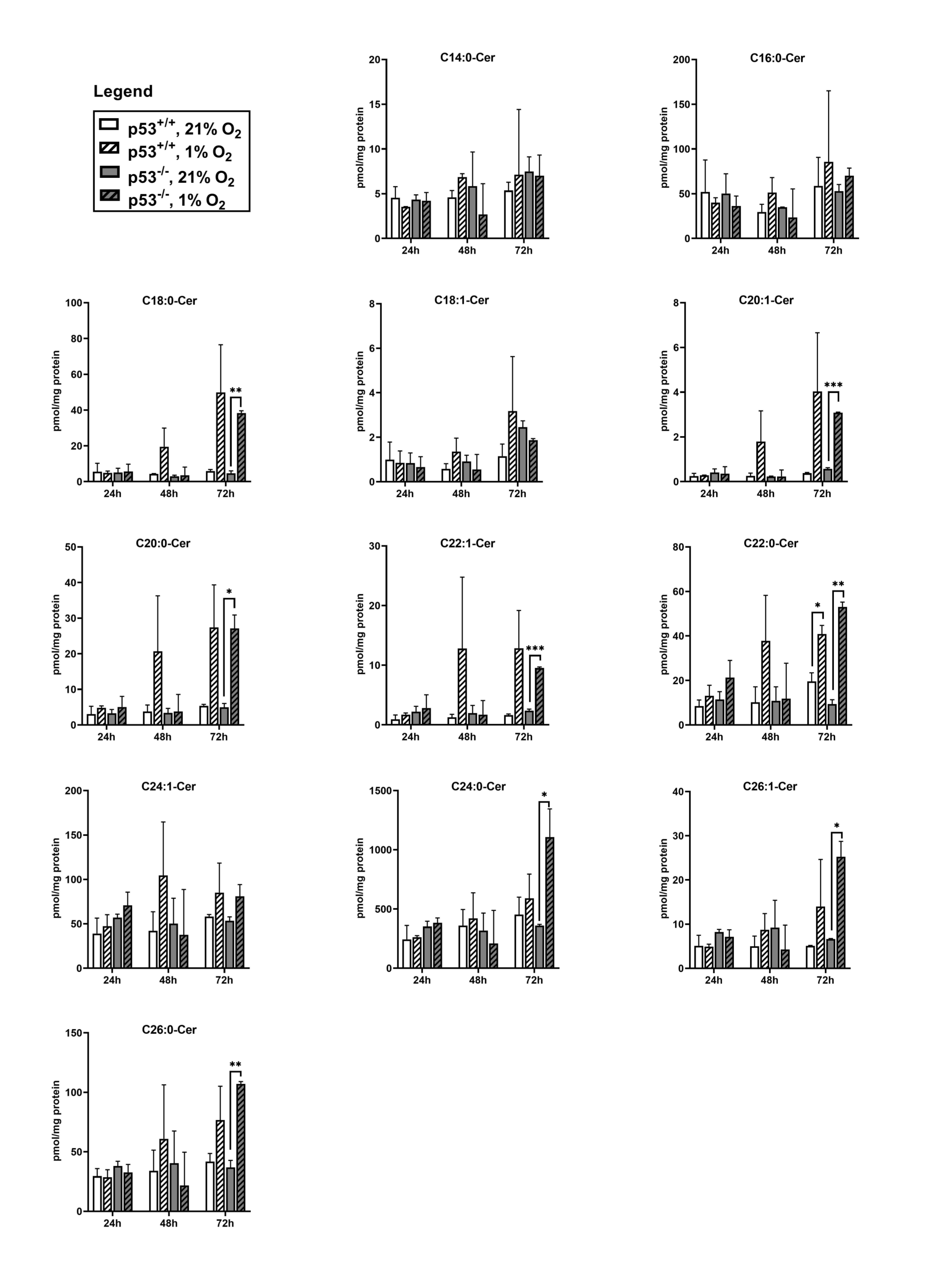

Supplement: S1 Fig — Different molecular species of ceramide were quantified by liquid chromatography-mass spectrometry and normalized to protein content (pmol ceramide/mg protein). Data is the average of two independent experiments ± S.D. *p < 0.05; **p < 0.01; ***p < 0.001. (TIF) [file pone.0340295.s001.tif]

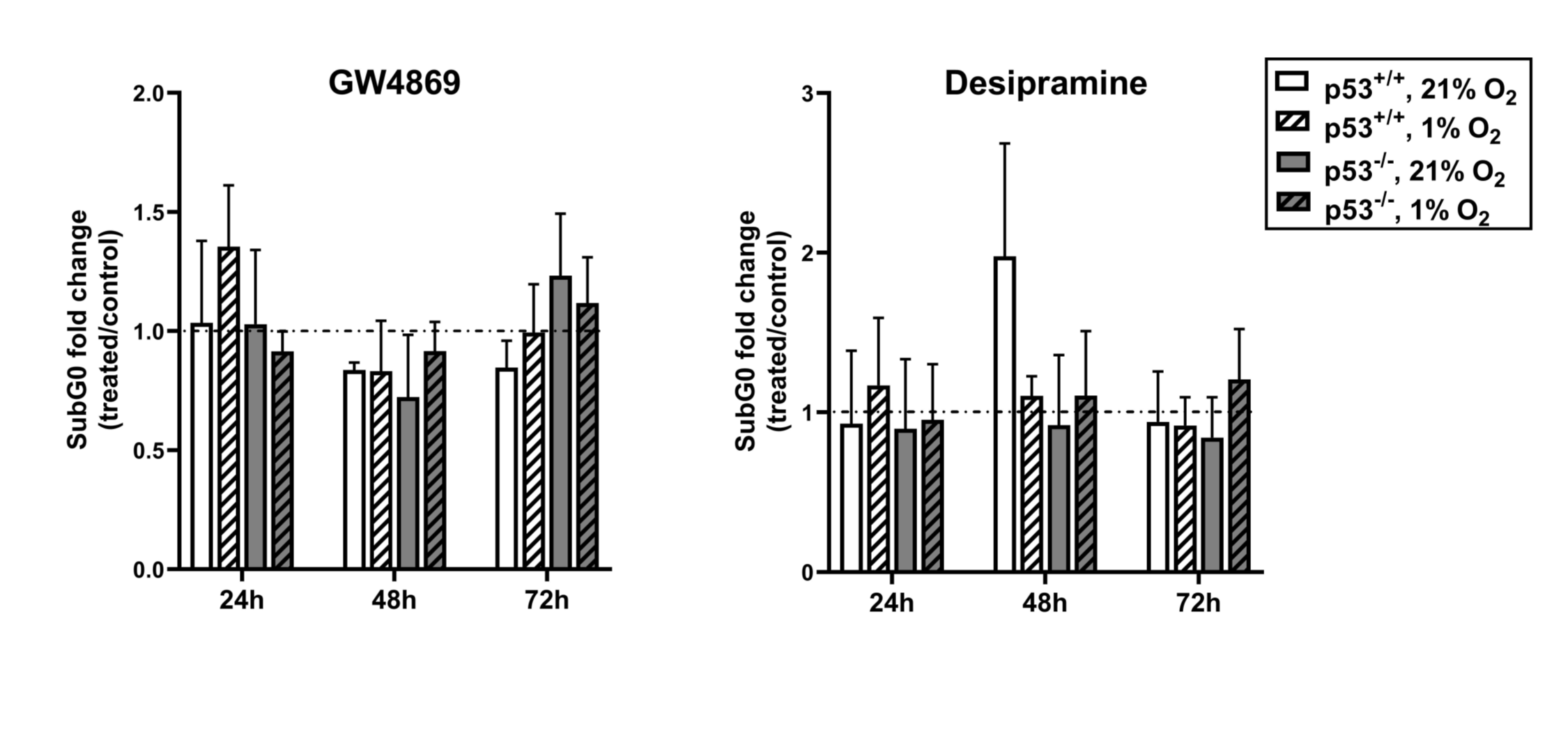

Supplement: S2 Fig — Fold change of the % of apoptotic cells in the GW4869-treated cells/control or in the Desipramine-treated cells/control. The percentage of cells in the subG0 phase of the cell cycle was quantified by flow cytometry. Values represent the ratios of the percentages obtained from GW4869-treated cells or Desipramine-treated cells over their vehicle-treated control. Each column represents the mean ± S.D. of the ratios calculated from three independent experiments. (TIF) [file pone.0340295.s002.tif]

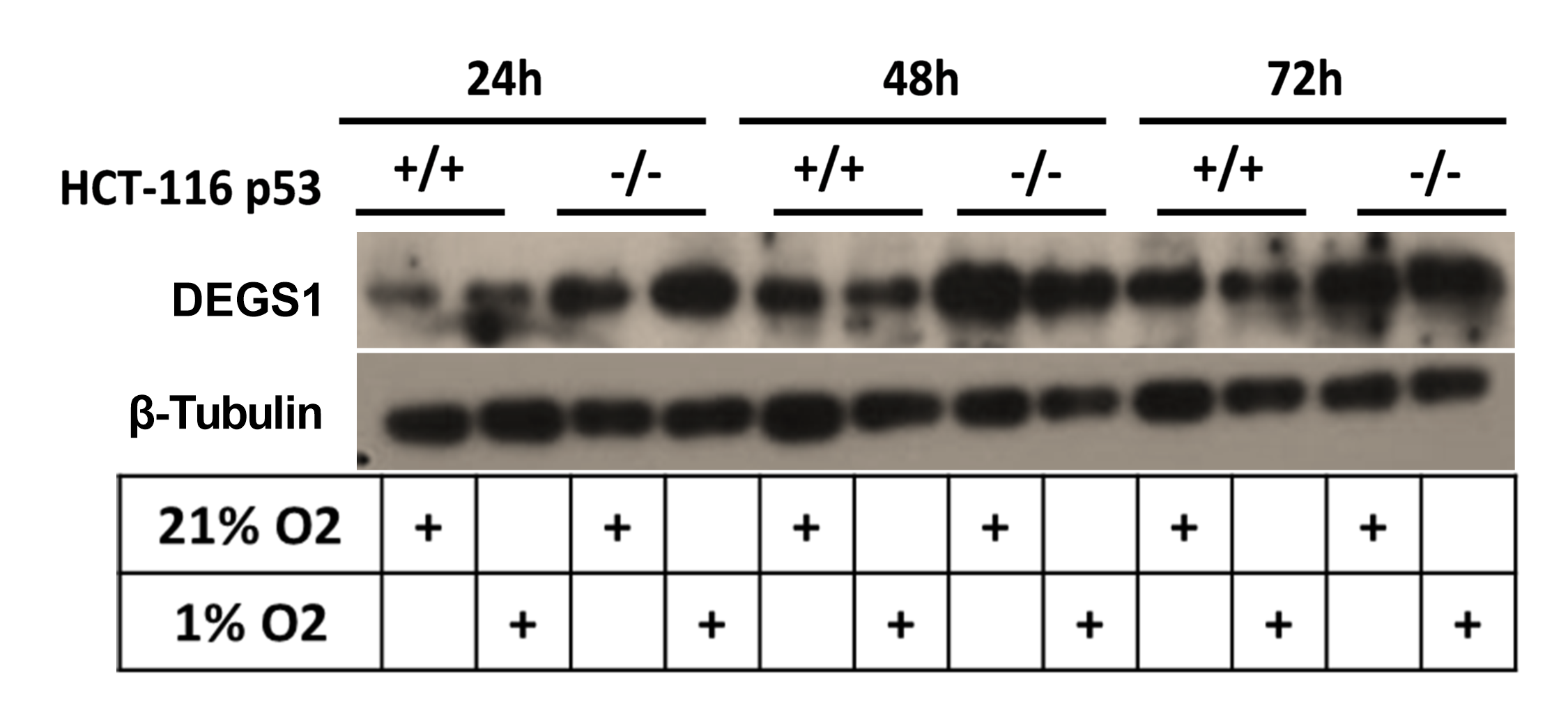

Supplement: S3 Fig — DEGS1 protein levels were assessed by western blot after 24, 48 and 72 hours of hypoxia. β-tubulin was used as a loading control (additional blot). (TIF) [file pone.0340295.s003.tif]
